# Transcranial Magnetic Stimulation and Dual Pathology: An Integrative Protocol

**DOI:** 10.1192/j.eurpsy.2024.326

**Published:** 2024-08-27

**Authors:** A. Moleon, P. Alvarez de Toledo, M. Martín-Bejarano, J. Narbona

**Affiliations:** Instituto Andaluz de Salud Cerebral, Sevilla, Spain

## Abstract

**Introduction:**

Dual pathology, characterized by the simultaneous presence of substance use disorders and psychiatric disorders, is a topic of growing interest in the scientific community. In particular, obsessive-compulsive disorder (OCD) is a common comorbid psychiatric condition in patients with substance use disorders.

**Objectives:**

To evaluate the efficacy of rTMS on comorbid disorder symptoms by applying specific protocols for OCD and substance use disorder in a clinical case of dual pathology.

**Methods:**

Case Description: A 36-year-old male diagnosed with OCD and habitual cocaine use (an average of 6 times per month). Previous unsuccessful attempts to quit substance use. Undergoing psychotherapy and psychopharmacological treatment for OCD since the age of 22 with no significant clinical improvement.

Methodology: The severity of OCD was quantified before and after the intervention using the Yale-Brown Obsessive Compulsive Scale (YBOCS). To assess addictive behavior, the Maudsley Addiction Profile (MAP) was used. During the intervention period, the occurrence of substance use was recorded based on the patient’s and family members’ reports. The intervention involved the administration of an rTMS protocol tailored to the specific case, consisting of the simultaneous application, using a double-cone coil, of rTMS at 20Hz over the right dorsomedial prefrontal cortex (DMPFC) at an intensity of 100% of the resting motor threshold (RMT) to treat OCD symptoms, followed by intermittent theta burst stimulation (TBS) over the left DMPFC at an intensity of 120% of the RMT to address substance addiction. The patient received a total of 30 sessions at a rate of one session per day, five days a week, for six weeks.

**Results:**

Results: The results showed an improvement in the total score on the YBOCS scale, decreasing from a value of 26 in the pre-intervention assessment to 16 in the post-intervention assessment, representing a reduction of more than 35% from pre- to post-intervention, meeting response criteria. Thus, there was a decrease in both obsessive and compulsive symptoms, with reduced associated distress and increased control. Additionally, throughout the intervention, there was a gradual decrease in substance use, decreasing from an average of 6 monthly instances before treatment initiation to a total of 1 in the month the treatment ended.

**Conclusions:**

Conclusions: This unique case study represents a therapeutic window for the treatment of patients with comorbid disorders, demonstrating promising preliminary benefits of the combined rTMS intervention for both conditions, especially in the field of addictions.

Keywords: rTMS, neuromodulation, obsessive-compulsive disorder, addictions

**Disclosure of Interest:**

None Declared

